# Silk Powder from Cocoons and Woven Fabric as a Potential Bio-Modifier

**DOI:** 10.3390/ma14226919

**Published:** 2021-11-16

**Authors:** Anna Baranowska-Korczyc, Andrzej Hudecki, Irena Kamińska, Małgorzata Cieślak

**Affiliations:** 1 Department of Chemical Textiles Technologies, Łukasiewicz Research Network-Textile Research Institute, 5/15 Brzezinska Street, 92-103 Lodz, Poland; irena.kaminska@iw.lukasiewicz.gov.pl (I.K.); malgorzata.cieslak@iw.lukasiewicz.gov.pl (M.C.); 2 Department of Functional Materials, Łukasiewicz Research Network-Institute of Non-Ferrous Metals, 5 Sowińskiego Street, 44-100 Gliwice, Poland; andrzej.hudecki@imn.gliwice.pl

**Keywords:** silk, cryogenic milling, fibroin, bio-modifier, textiles, degumming

## Abstract

Silk, as a protein fiber characterized by high biocompatibility, biodegradability, and low toxicity, is mainly used as textile structures for various purposes, including for biological applications. The key issue for unlimited silk applicability as a modifier is to prepare its relevant form to cover or introduce to other materials. This study presents silk powder fabrication from *Bombyx mori* cocoons and non-dyed silk woven fabric through cryogenic milling. The cocoons were milled before and after the degumming process to obtain powders from raw structures and pure fibroin. The powder morphology and composition were analyzed using scanning electron microscopy and energy dispersive spectroscopy. The influence of the milling on the silk structure was studied using infrared and Raman spectroscopies, indicating that silk powders retained dominant β-sheet structure. The powders were also analyzed by differential scanning calorimetry and thermogravimetric techniques. The thermal endothermic peak and onset temperature characteristic for silk decomposition shifted to the lower values for all powders, indicating less thermal stability. However, the process was found to be an efficient way to obtain silk powders. The new milled form of silk can allow its introduction into different matrices or form coatings without using any harsh solvents, enriching them with new features and make more biologically friendly.

## 1. Introduction

Silk has been extensively studied as one of the most promising natural-origin materials for biotechnological and biomedical purposes [1]. Due to its unique properties, such as remarkable strength and toughness, high thermal stability, biocompatibility, biodegradability, low toxicity, and textile technological importance, it has been applied as a drug delivery system, scaffolds for tissue engineering, or for designing biosensors and wearable electronics [2]. Silk is a biopolymer composed of two unique proteins, fibroin and sericin. Silk in *Bombyx mori* (*B. mori*) cocoons is mostly formed by fibroin, about 70 to 80 wt.%, which after degumming is commonly used in the textile industry and in medicine [3]. Fibroin exhibits high mechanical resistance due to antiparallel alignment of β-sheets in its protein secondary structure, hydrophilic and hydrophobic blocks in a semicrystalline polymer matrix, self-cooling ability, and a lack of inflammatory responses in humans [4]. Sericin is also applied for biomedical systems due to its high moisture, oxidation, and protection against UV radiation [5]. The excellent properties of both silk proteins mean that they are processed in many ways and forms including in vivo modification, regeneration, or post-treatments. Silk fibers can be dissolved, doped with different nanostructures, or form a base for various dimensionality systems, such as films, microspheres, or electrospun fibers [1]. Fibroin and sericin have been used mainly to improve the physical properties of various materials as well as to enhance their biocompatibility.

However, most silk modification methods such as spin or dip coating and electrospinning, need to use harsh solvents (trifluoroacetic acid, lithium bromide, methanol, or formic acid) which limited the applicability of silk proteins [2,6]. The key issue for the unlimited use of silk as a bio-modifier is to prepare its relevant form to cover or introduce to other materials using physical techniques instead of wet chemistry methods. There are some reports which introduce milling techniques as a facile way for processing natural silk fibroin. The commercial mulberry, muga, and eri silk were degummed and pulverized to ultrafine powder using rotary and ball milling [7]. The fibroin from eri silk cocoon after removing sericin was also converted to powder through attritor and jet milling [8]. Raw *B. mori* silk was also degummed and treated by mechanical milling to obtain fibroin powder for efficient removal of toxic dyes from an aqueous solution [9]. The above reports were focused on fibroin pulverization, whereas the milling of raw cocoons can result in combining the advantageous properties of both proteins in the fibroin/sericin powder.

This study presents pulverization of different silk-based materials and includes not only fibroin but also raw *B. mori* cocoons and woven fabric. The raw cocoons were grinding to obtain powder composed of fibroin and sericin for the combining of the advantageous properties of both proteins. The possibility of silk powder formation from previously woven fabric was evaluated. The process of cryogenic milling was selected as a method that does not generate heat since high temperatures can harmfully influence the protein structure and properties. The morphology and chemical composition of the powders obtained from three different sources were studied using, respectively, scanning electron microscopy (SEM) and energy dispersive spectroscopy (EDS). To evaluate the efficiency of the degumming process and silk structure, Raman and infrared spectroscopies were implemented in the study. It was found that sericin was effectively removed from the raw cocoon in the case of fibroin samples and all powders retain a dominant β-sheet structure. The thermal properties of all samples were analyzed using differential scanning calorimetry (DSC) and thermogravimetry/derivative thermogravimetry (TG/DTG). The thermal degradation process started at lower temperatures for all powders in comparison to the source materials. The thermal endothermic peak characteristic for silk decomposition also shifted to the lower values for all powders. Despite the slightly lower thermal stability caused by the fragmentation of the material, it has been shown that low-temperature silk grinding is an effective method of obtaining silk powders composed of sericin and fibroin mixture (or only fibroin). The protein powders can be introduced to the various systems without applying harsh solvents for various biological applications.

## 2. Materials and Methods

### 2.1. Silk Cocoon Degumming and Cryogenic Milling

*Bombyx mori* silk cocoons were obtained from a Chinese (Shenzhen) market supplier. Non-dyed silk woven fabric (mass per unit area of 70 g/m^2^, thickness of 0.2 mm, linear mass of yarns of 37 dtex) was obtained from Łukasiewicz Research Network-Textile Research Institute resources. The raw cocoons were cut to small pieces and degummed in an aqueous solution of 0.02 M sodium carbonate (Na_2_CO_3_, Sigma Aldrich, Poznań, Poland). 0.4 g of the cocoons were immersed in 200 mL of Na_2_CO_3_ for 40 min at 100 °C. Then, silk fibroin was rinsed three times in cold distilled water and dried for 24 h at 40 °C. To determine the fibroin content, the raw cocoons and cocoons after the degumming process were dried (24 h at 40 °C) and immediately weighted.

**Figure 1:**
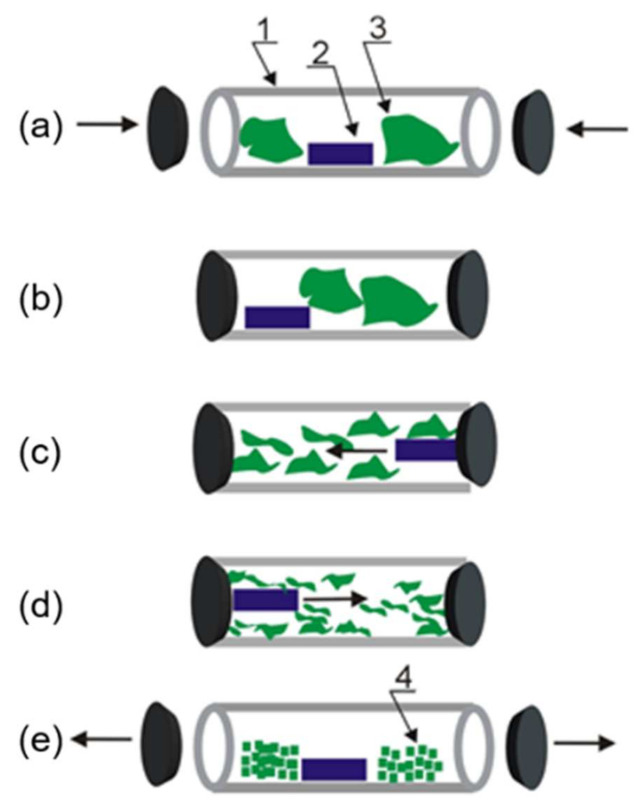
Scheme of the subsequent stages of milling process: (**a**) loading the sample into the vial, (**b**) closing the vial, (**c**) starting the grinding process, (**c**,**d**) sample grinding by magnetically shuttling impactor oscillating back and forth along the vial, and (**e**) removing powder from the vial. The numbers in the diagram label sequentially (1) the vial, (2) impactor, (3) sample, and (4) powder.

The milling process was carried out using cryogenic grinder (SPEX^®^ SamplePrep, Metuchen, NJ, USA) of 6875D model (Figure 1). Before the milling process, the raw cocoons, cocoons after the degumming process (silk fibroin), and silk woven fabric were dried in the Christ Alpha 2-4LD dryer (Martin Christ Gefriertrocknungsanlagen GmbH, Osterode am Harz, Germany) for 24 h at 30 °C. The samples of weight about 4 g were placed in polycarbonate vials of 100 mL volume with magnetically shuttling impactors. The samples before milling were immersed in liquid nitrogen in cryogenic milling vials for 60 min to freeze them. The milling process was repeated five times for 5 min with 2-min breaks without stirring to decrease the temperature in the chamber to the starting value and keep the sample frozen. The vial with the sample was continuously immersed in liquid nitrogen to maintain cryogenic temperatures. The single milling cycle was composed of 15 impactor blows. After the milling process, the samples were dried again to remove the excess water.

### 2.2. Characterization of Silk-Based Powders

The morphology of the samples was analyzed by using SEM VEGA 3 TESCAN (Tescan Osay Holding, Brno, Czech Republic) at an accelerating voltage of 20 kV. The samples for SEM analysis were covered with a thin gold layer of about 3 nm. Chemical composition was investigated by EDS analysis (INCA Energy X-ray energy dispersion spectrometer, Oxford Instruments, Abingdon, UK) of three different area of 0.2 mm^2^, with an accelerating voltage of 20 kV and pressure of 20 Pa. The statistical analysis for EDS measurements were performed using the INCA energy software 450 (version 4.15). Raman spectra of the samples were obtained by using an inVia Renishaw Raman Microscopy System (Renishaw, Wotton-under-Edge, UK) with a 50× microscope objective (LEICA, Wetzlar, Germany). The excitation source was a laser with a wavelength of 785 nm. Each Raman spectrum was obtained with four accumulations in the range of 600 to 2000 cm^−1^ corrected by the WiRE™5.3 software. The Raman spectra were presented without normalization. The analysis of the band intensity was based on the comparison of selected bands within one spectrum and then this intensity ratio was compared for all samples. The Fourier transformed infrared (FTIR) absorption spectra of silk sample were recorded using BRUKER Vertex 70 FTIR spectrometer with diamond ATR Golden Gate adapter(Bruker, Bremen, Germany) spectrometer, in a spectral range of 600 cm^−1^ to 4000 cm^−1^ with a resolution of 4 cm^−1^. The Raman and infrared spectra were collected at least three replicates for each sample.

The thermal properties were studied using DSC 204 F1 Phoenix calorimeter, (Netzsch, Selb, Germany) and TG 209F1 Libra (Netzsch, Selb, Germany) techniques. The thermal measurements were performed at least three replicates for each sample. The samples with a weight of about 4 mg were placed in a ceramic crucible with a volume of 85 μL and heated with a rate of 10 °C min^−1^ under nitrogen flow of 25 mL min^−1^ in the temperature range of 20–600 °C and 20–800 °C for DSC and TG/DTG analysis, respectively. Student’s *t*-test was used for comparison thermal properties between the samples before and after milling process.

## 3. Results and Discussion

### 3.1. Powder Formation and SEM/EDS Studies

**Figure 2:**
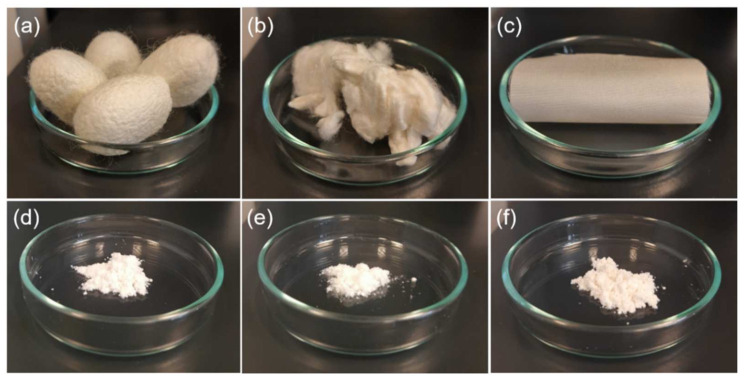
(**a**) Raw and (**b**) degummed silk cocoons, (**c**) silk fabric, and silk powders from (**d**) raw cocoons, (**e**) degummed cocoons, and (**f**) silk fabric.

In this study, silk powder was obtained by cryogenic milling (Figure 1) from raw silk cocoons, cocoons after the degumming (that is, fibroin), and woven silk textile (Figure 2a–c). The milling process allowed the silk processability without using harsh substances, such as trifluoroacetic acid, lithium bromide, or formic acid applied for fibroin coating formation or electrospinning [2,6]. The silk fibroin is characterized by high thermal stability with no clear thermal degradation below 200 °C [10]. However, the milling procedures often referred to as processes carried out “at room temperature” can cause a significant increase in temperature, for example even to 600 °C for planetary ball milling [11]. The recent study indicated also that native silk is stable only between −10 and 55 °C [12] due to sericin and fibroin additional temperature transition appearing, respectively, at about 45 and 65 °C prior to the main silk endothermic transition. The thermal denaturation was redefined for silk proteins as a loss of natural hydration shell, which results in structural reconfiguration in native material [12]. The low temperature was applied for the milling process, since silk cocoons are composed of natural fibers which need special treatment to avoid protein denaturation and changes in their structure and properties.

The milling process is a facile way allowed to form effectively a silk powder from raw and degummed cocoons and fabric (Figure 2d–f). The three materials were selected to evaluate the milling process of various silk-based materials and for further extension of the silk’s applicability. Previously, only the fibroin was ground and applied for different systems [7,8,10]. The raw cocoons were chosen for the study to obtain a powdered modifier composed of fibroin and sericin. Sericin from cocoons of *B. mori* has attracted growing attention since its unique properties were characterized in many reports. This included promotion of collagen production, anti-inflammatory activity, controlled drug-release, and antioxidant properties [13]. The accumulation of specific properties of fibroin and sericin can expand significantly the application range of silk powder as a bio-modifier. The silk textile structure was selected to study the possibility of using woven fabric to form a powder and its properties as a potential modifier. This approach is consistent with recycling strategy of textile waste. The milling process can allow one to obtain regenerated silk in a form of powder from waste of silk woven textile structures during their production and from reused materials as well as waste generated during the process of obtaining silk from cocoons.

**Figure 3:**
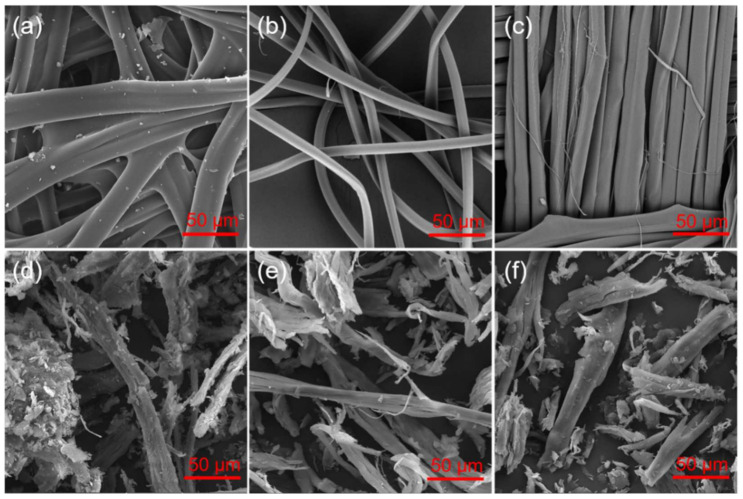
SEM images of (**a**) raw silk cocoons, (**b**) degummed silk cocoons, (**c**) silk fabric, and silk powders from (**d**) raw cocoons, (**e**) degummed cocoons, and (**f**) silk fabric.

The original materials significantly changed form due to the fragmentation of the samples. The morphology of the materials was also extensively modified. SEM images of the raw cocoons reveal typical structures of two fibroin filaments bonded together since they are originally extruded from two silkworm glands (Figure 3a). They remain together due to protein gum of sericin, visible on the fiber surface. The debris visible in the sample indicates the raw nature of the cocoons without applying any cleaning treatment. After the degumming process and removing sericin, the fibroin filaments are not bounded and their surface is smooth without additional components (Figure 3b). The silk fabric with plain weave contains the same yarn in warp and weft (Figure S1). The cryogenic milling caused fragmentation of the fibers of all original materials to a similar degree, the obtained fibroin filament segments are characterized by the size range of about 30 to 500 µm (Figure 3d–f). However, the process was slightly more efficient for the fabric sample, which shows the smallest fiber fragments of about 30–350 µm (Figure 3f). It indicates lower durability of this silk probably due to previous fabric manufacturing processes in comparison to raw and degummed cocoons.

EDS analysis revealed the main elements corresponding to silk chemical composition, such as carbon (C), nitrogen (N), and oxygen (O) for all samples. Moreover, raw cocoons contained trace amounts of calcium (Ca), sulfur (S), and potassium (K) (Table 1). Ca is one of the fingerprints of natural silk [14]. However other elements such as S, K, and also sodium (Na) and chloride (Cl) were previously noted in a trace amount in the composition of *B. mori* cocoons before the degumming process [15]. The cocoons after removing sericin and for silk fabric did not reveal the presence of the above-mentioned elements since they were clear away by rinsing in water after the degumming process. The degummed cocoons were only composed of C, N, and O. The trace amount of sodium (Na) was noted for cocoons without sericin and for silk fabric (Table 1) due to applying sodium carbonate as a degumming agent. EDS study confirmed the chemical composition of the source materials in comparison to other reports and indicated that the cryogenic milling process did not affect their composition significantly.

### 3.2. Raman Studies

**Figure 4:**
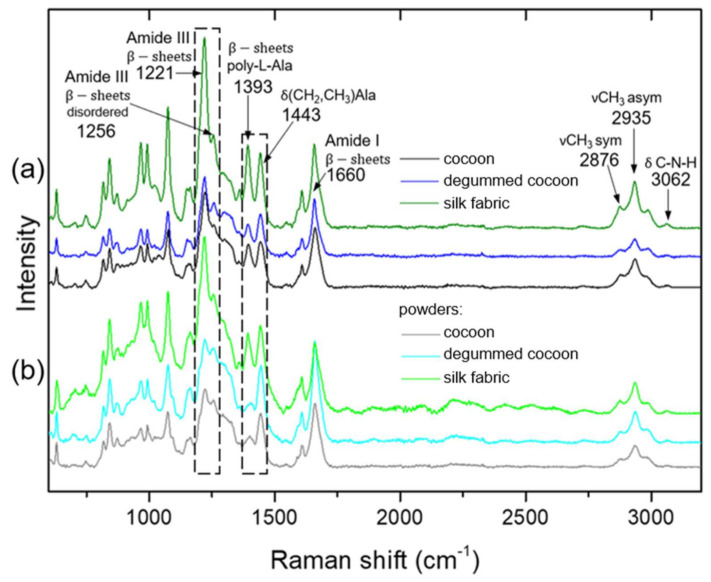
Raman spectra of (**a**) raw silk cocoons, degummed cocoons, and silk fabric; and (**b**) silk powders from raw cocoons, degummed cocoons, and silk fabric.

To study the protein conformation and the influence of cryogenic milling on silk materials Raman spectroscopy analysis were implemented to the report. Figure 4 and Figure S2 show Raman spectra of all samples before and after the milling process. It is clearly visible that all spectra revealed the same bands responsible for specific assignments of silk native structure, indicating no significant influence of degumming and cryogenic milling on the fibroin structure. The peak at 1660 cm^−1^ related to the amide I of β-sheets C=O stretching [16] is present in all spectra at comparable intensity and the same value of Raman shift. β-sheets structure of amine III is also visible for all samples as the bands at 1221 and 1256 cm^−1^ [17]. The intensity of the band at 1221 cm^−1^ decreased for the powders, especially for raw and degummed cocoons, in comparison to 1256 cm^−1^ band related to disordered form of β-sheets amide III. It indicates that milling process resulted in the increase the number of regions of the protein chain, which do not form regular secondary structure of amide III. However, the presence of the bands corresponding to β-sheets of amide I and amine III indicate the crystalized regions of fibroin regardless to milling process. The bands at 3062, 2935, and 2876 cm^−1^ related, respectively, to C-N-H bending, CH_3_ asymmetric stretching, and CH_3_ asymmetric stretching visible in all Raman spectra demonstrate that fibroin structure were well preserved [17].

The changes in the intensity of other selected bands were noted. The band at 1393 cm^−1^ originating from β-sheet parts of poly-L-alanine [18] decreased after the milling process in comparison to the band at 1443 cm^−1^ corresponding to CH_2_, CH_3_ bending in alanine [19]. It indicates that cryogenic milling process effectively cut the long aminoacidic sequences, such as polyalanine to smaller parts. The spectra of raw cocoon and degummed samples are very similar due to the fact that Raman spectroscopy does not clearly distinguish sericin presence. It was found previously that the Raman intensity of the sericin was found to be negligible [20].

### 3.3. Fourier Transformed Infrared (FTIR) Analysis

**Figure 5:**
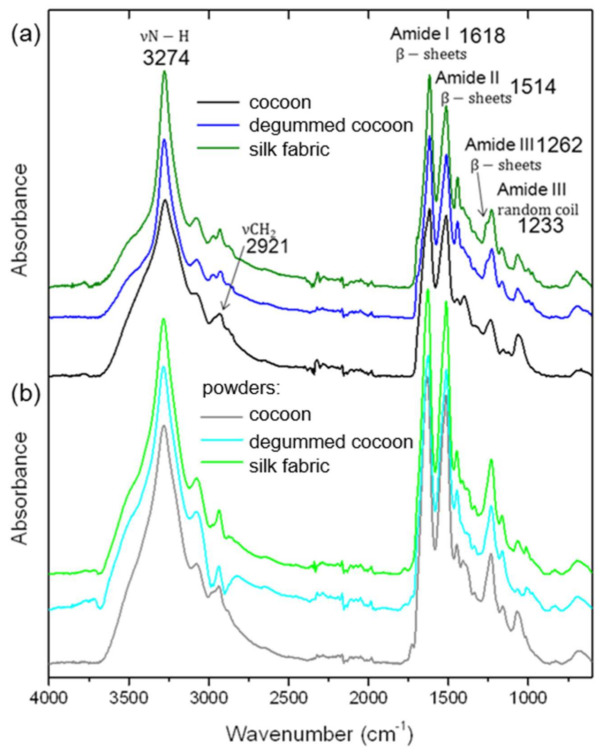
Infrared absorption spectra of (**a**) raw, and degummed silk cocoons, silk fabric, and (**b**) silk powders from raw cocoons, degummed cocoons, and silk fabric.

**Figure 6:**
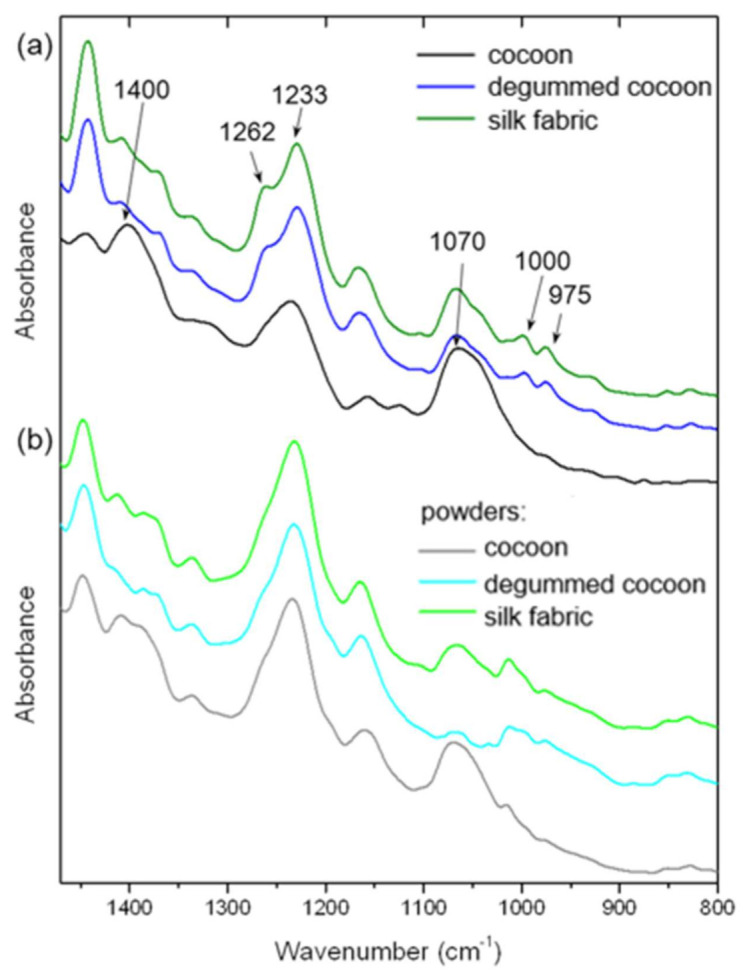
Infrared absorption spectra of (**a**) raw, and degummed silk cocoons, silk fabric and (**b**) silk powders from raw cocoons, degummed cocoons, and silk fabric.

The infrared spectroscopy studies were applied as complementary data to Raman spectroscopy results. FTIR analysis is a valuable tool for the evaluation of silk fibroin secondary structure and individual components of silk samples, including sericin. The characteristic infrared bands indicate different conformations of silk protein structure. FTIR spectra were measured for raw and degummed silk cocoons as well as silk fabric. Moreover, the spectra were collected and analyzed for all of the above-mentioned samples after the cryogenic milling process (Figure 5 and Figure S3). The main peaks characteristic of silk fibroin were noted for raw cocoons, degummed cocoons, and fabric at the maximum of 1618, 1514, and 1262 cm^−1^ indicating β-sheets crystallites of, respectively, amide I, II, and III (Figure 5a) [21]. The amides bands connected with β-sheets conformation did not change after the degumming process indicating preserving the silk structure. The band at a maximum of 1233 cm^−1^ corresponding to a random coil of amide III [22] also did not change for all samples indicating that this structure of fibroin parts remained unchanged. In Figure 5a, the band at about 3274 cm^−1^ revealed a broad peak for raw cocoons indicating the contribution of sericin characterized with a more random structure than high crystalline fibroin with stacked β-sheets. After the degumming process and for silk fabric, the band is narrower, demonstrating an efficient sericin removing process [23]. The evaluation of the degumming process is also possible by examination of the presence of different peaks, for example at 1400 cm^−1^ connected with sericin and present only in raw silk cocoon sample (Figure 6a and Figure S4) [23]. Moreover, the sample without sericin or with its little amount shows two bands at 975 and 1000 cm^−1^ indicating, respectively, an abundance of -glycine-alanine- and -glycine-glycine- fragments in polypeptide chain (Figure 6 and Figure S4) [23]. The evidence of sericin presence before the degumming process is visible in the infrared peak at 1070 cm^−1^, whose intensity is relatively higher for the sample not chemically treated and decreases with increasing the content of the fibroin in the silk-based materials [23]. The low-intensity peak at 2921 cm^−1^ responsible for stretching vibration of CH_2_ is only observed in raw silk (Figure 5a and Figure S3) due to the sericin presence [24]. It was also noted that after the degumming process a band with the maximum at 1262 cm^−1^ of amine III band in comparison of 1233 cm^−1^ band became more evident after removing sericin due to the increasing content of β-sheet crystallites of fibroin in the sample (Figure 6a and Figure S4). The intensity of the band increases with increasing degumming time and ratio [25]. After the milling process, the peak at 1262 cm^−1^ is not so apparent due to an increase in the intensity of 1233 cm^−1^ band related to non-oriented β-sheet arrangement in silk fibroin of amide III.

The maxima of the main peaks from the infrared fingerprint region for amide II and amide III did not shift for all powders regardless of the silk source. The maxima of the amide I band slightly shifted to the higher values of 1632, 1626, and 1629 cm^−1^, respectively, for raw, degummed cocoons, and fabric indicating rearrangement in β-sheet conformation involving C=O stretching in tyrosine. The presence of the three main amides bands after the milling process demonstrates that β-sheets crystallites parts of fibroin were preserved for all powders. Moreover, bands for a random coil of amide III at 1233 cm^−1^ were also noticeable after milling indicating that amorphous parts of the fibroin also did not significantly change. The main infrared peaks of the powders indicate that the main secondary conformation of β-sheets and random coil for silk was retained after degumming and milling processes. Additionally, infrared spectra do not show any others picks besides characteristic silk bands that confirmed the native secondary conformation of silk protein fibers was preserved.

### 3.4. Thermal Analysis

**Figure 7:**
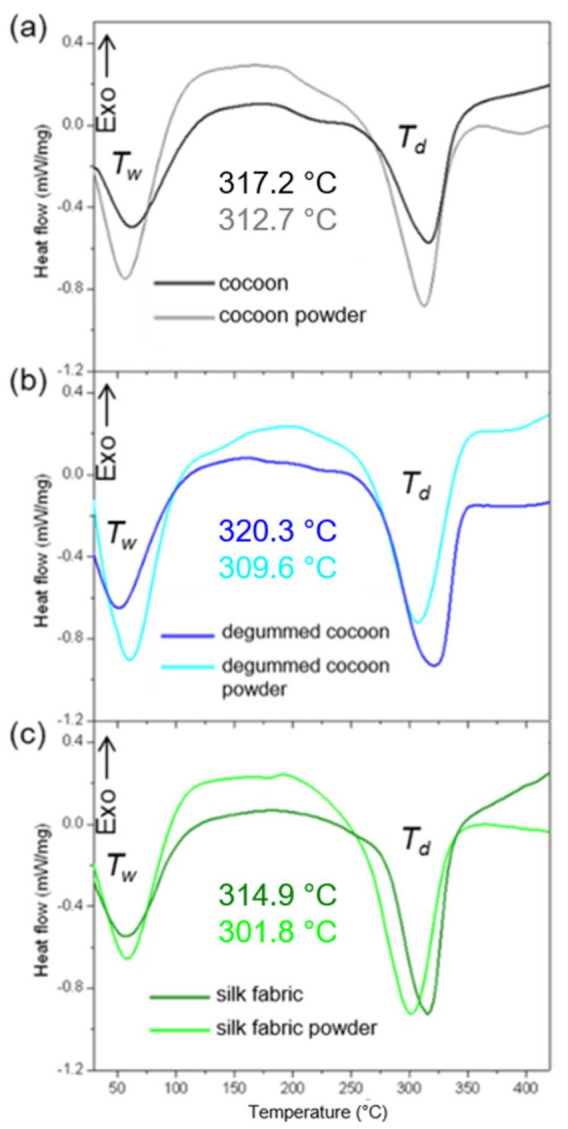
DSC thermograms of (**a**) raw and (**b**) degummed silk cocoons, and (**c**) silk fabric, and silk powders from (**a**) raw cocoons, (**b**) degummed cocoons, and (**c**) silk fabric.

The DSC analysis of the silk samples was also carried out to define their thermal properties and compered source silk materials to their powdered form (Figure 7). Figure 7 shows thermograms of raw silk cocoons, degummed cocoons, and silk fabric, before and after the milling process. The detailed results are given in Table S1. A broad endothermic peak (denoted as Tw) cantered around 60 °C correspond to water loss was noted for all samples. The samples were stored at the same conditions characterized by humidity of 50% and temperature of 23 ± 2 °C. The maximum of the peak for silk-based materials before milling slightly varies and appears at the highest temperature of 62 °C for raw cocoons since removing water molecules from silk fiber entwined is more difficult than from fabric. Tw peak maximum for degummed cocoons is noted at about 52 °C due to the fact that they are previously dried after sericin rinsing in an aqueous solution. Based on the dry mass of raw cocoons before and after degumming it was calculated that fibroin content is about 73 wt.%, which is consistent with other reports [3]. The endothermic Tw peak for powders is centered about 60 °C for all milled samples, indicating water absorption by the powders and its evaporation during the measurements.

DSC analysis of raw cocoons indicated their thermal decomposition in the temperature range of 270.9 to 337.3 °C and endothermic peak at about 317.2 °C, denoted as T_d_ and corresponded to degradation of β-sheet structures [26,27]. This temperature range changed slightly after the raw cocoon milling process and was noted from 268.9 °C to 335.3 °C. The endothermic peak was shifted by 4.5 °C to a lower value and was estimated to be about 312.7 °C indicating lower thermal stability of the cocoons after the milling process due to breakage of amide bonds and non-oriented β-sheet arrangement in the silk fibroin structure [28]. This phenomenon was also noted for the cocoons without sericin; the onset and end temperatures for degummed samples were noted, respectively, at 272.8 °C and 342.9 °C before and 263.4 °C and 334.8 °C after the milling process. The endothermic peak was shifted by 10.7 °C from a value of 320.3 °C to 309.6 °C due to decreasing thermal degradation temperature. The silk fabric thermal properties revealed the lowest thermal stability before as well as after the grinding process. Even though the decomposition range of the fabric is similar to raw cocoon values of 274.0 °C to 336.1 °C, the maximum of the endothermic peak was noted at 314.9 °C. After the milling process of silk fabric, the thermal decomposition process was carried out at significantly lower temperature values. The onset and end temperatures were measured to be 258.6 °C and 334.0 °C, respectively. The maximum of the endothermic peak shifted about 12 °C to the value of 301.8 °C demonstrating the significant impact of the grinding process on the thermal properties of silk fabric and making them lower thermally resistant. The degradation enthalpy value (ΔH_deg_) for raw cocoons, after removing sericin and for silk fabric was determined to be 228.5, 278.9, and 331.9 J/g, respectively. ΔH_deg_ values after the milling process were estimated to be 257.7, 286.1, and 333.7 J/g, respectively, for the powders from raw cocoons, degummed cocoons, and fabric. The heat of the thermal decomposition of the silk samples after grinding increased slightly, indicating that the thermal stability of the samples has not changed significantly, while the onset, end, and endothermic peak maximum were shifted to the lower values. To evaluate the significance level of the thermal decomposition temperatures of the samples before and after the milling process the statistical *t*-test was performed. It was found that significant differences (*p* < 0.05) were noted for endothermic peak position for milled raw cocoons, end temperature of the process for degummed cocoons, and the maximum of the peak and end temperature for silk fabric.

**Figure 8:**
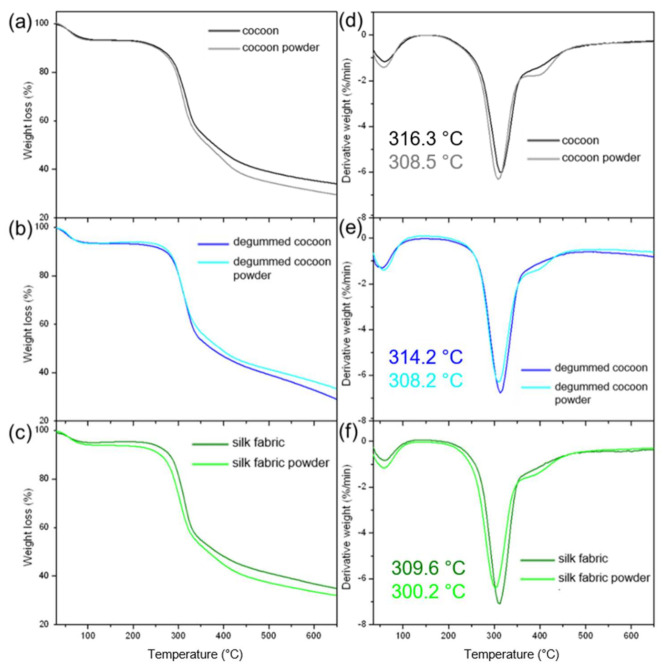
(**a**–**c**) TG and (**d**–**f**) DTG thermograms of (**a**,**d**) raw and (**b**,**e**) degummed silk cocoons, and (**c**,**f**) silk fabric, and silk powders from (**a**,**d**) raw cocoons, (**b**,**e**) degummed cocoons, and (**c**,**f**) silk fabric.

The thermogravimetric analysis (Figure 8) revealed an initial weight loss in all samples at about 60 °C related to water evaporation, which is consistent with the DSC results. The main process in the studied range of temperatures is connected to heat absorption for the decomposition of silk-based materials. In the region from 250 °C to 350 °C (Figure 8b), the rapid increase in weight loss is observed due to the breakage of peptide bonds and reduction of intermolecular interactions [29]. The thermal decomposition of raw and degummed cocoons and their powders started at about 281.0 °C. The process onset temperature for silk fabric and its powdered form was lower by about 2 °C indicating lower resistance to heat for silk fabric and its powder. The detailed results are given in Table S2. It was found that the milling process not affected significantly the starting temperature of the thermal decomposition process. The maximum of the thermal decomposition peak was noted at 316.3, 314.2, and 309.6 °C for raw and degummed cocoons and woven fabric, respectively. The values were characteristic for silk-based materials [30] and indicated the highest thermal resistivity for raw cocoons and the lowest one for silk fabric. The earlier degradation temperature for the fabric may be attributed to a decrease of molecular weight of peptide chains due to previous manufacturing processes of silk fabric [29]. The thermal degradation peak after the milling process was found to be about 308.5, 308.2, and 300.2 °C, respectively, for raw cocoons, degummed cocoons, and fabric. The peak was shifted slightly by about 8 °C (7.8 ± 0.5 °C), 6 °C (6.0 ± 1.1 °C), and 9 °C (9.4 ± 1.2 °C) to lower temperatures demonstrate that the milling process makes silk less thermally resistant material. To analyze the relevance of the milling on the decomposition process the significance level was estimated (*t*-test) for the thermal decomposition temperatures of the samples before and after the grinding process. The significant changes (*p* < 0.05) were indicated for end temperature of decomposition process raw cocoon and its powder; onset, end temperatures, and a maximum of the peak for degummed cocoon in comparisons to its powder; and end temperature and peak position for silk fabric and its powder. The thermal processes were significantly influenced by the milling process. However, the maximum of the thermal degradation peak was noted still above 300 °C indicating that the high thermal stability of the silk was retained for all powders.

## 4. Conclusions

The study demonstrates an easy method of obtaining silk powder from raw cocoons, degummed cocoons, and silk woven fabric. The powder from raw cocoons is composed of fibroin and sericin, while degummed cocoons and silk fabric are ground into fibroin powder. The silk-based materials are pulverized using cryogenic milling. The low-temperature process is applied to minimize the changes in secondary silk conformation. FTIR and Raman spectroscopies revealed that the powders retained dominant β-sheet structure and some random coil fragments. The rearrangement was only noted in the β-sheet amide III part and fragmentation of poly-L-Ala fragments due to the milling process. The thermal properties of the powders also show characteristic decomposition for silk materials. The thermal endothermic peak and onset temperature shifted to the lower values for all powders indicating their less thermal stability, especially silk woven fabric and its powder. However, the process of sericin/fibroin and fibroin powder formation is found to be efficient and beneficial due to mild temperature conditions and no use harsh solvents. The powders can extend silk applications as a bio-modifier of various systems due to easy introduction into different matrices or forming coating to enrich them with new properties and make them biocompatible.

## Figures and Tables

**Table 1:** Results of EDS analysis of raw silk cocoons, degummed cocoons, silk fabric, and their powders.

	C (wt.%)	N (wt.%)	O (wt.%)	Ca (wt.%)	S (wt.%)	K (wt.%)	Na (wt.%)
raw cocoons	44.5 ± 0.3	16.9 ± 0.3	37.6 ± 0.5	0.6 ± 0.1	~0.2	~0.2	
raw cocoons-**powder**	46.3 ± 0.6	17.5 ± 0.5	35.4 ± 0.5	~0.3	~0.1	~0.3	
degummed cocoons	52.4 ± 0.6	19.7 ± 0.3	27.6 ± 0.5				~0.2
degummed cocoons-**powder**	48.8 ± 0.1	20.6 ± 0.2	30.5 ± 0.1				~0.1
silk woven fabric	48.8 ± 0.4	20.9 ± 0.4	30.2 ± 0.3				~0.3
silk woven fabric-**powder**	51.9 ± 0.7	19.0 ± 0.4	28.9 ± 0.3				~0.3

## Data Availability

The data presented in this study are available on request from the corresponding author.

## Supplementary Materials

The following are available online at https://www.mdpi.com/article/10.3390/ma14226919/s1. Figure S1: SEM images of (a) the top view, (b) cross-sectional view, and along the fibers of silk woven fabric; Figure S2: Raman spectra of (a) raw silk cocoons, (b) degummed cocoons, and (c) silk fabric, and silk powders from (a) raw cocoons, (b) degummed cocoons, and (c) silk fabric; Figure S3: Infrared absorption spectra of (a) raw, and (b) degummed silk cocoons, (c) silk fabric, and silk powders from (a) raw cocoons, (b) degummed cocoons, and (c) silk fabric; Figure S4: Infrared absorption spectra of (a) raw, and (b) degummed silk cocoons, (c) silk fabric, and silk powders from (a) raw cocoons, (b) degummed cocoons, and (c) silk fabric; Table S1: The onset, and end temperatures, peak (T_d_) of degradation process, and degradation enthalpy value (ΔH_deg_) obtained from DSC analysis for raw, degummed silk cocoons and silk woven textile before and after cryogenic milling; Table S2: The onset, and end temperatures, and peak of degradation process, obtained from TG/DTG analysis for raw, degummed silk cocoons and silk woven textile before and after cryogenic milling.
