# Supplementary material for: Silk Powder from Cocoons and Woven Fabric as a Potential Bio-Modifier
Source: Materials (Basel). 2021 Nov 16;14(22):6919. doi: 10.3390/ma14226919 (PMC8624342; doi:10.3390/ma14226919)
Supplement: Supplementary file 1 [file materials-14-06919-s001.zip › materials-1419096-supplementary.pdf]

Supplementary Materials

Silk Powder From Cocoons and Woven Fabric as a Potential Bio-Modifier

Anna Baranowska-Korczyc [1], Andrzej Hudecki [2], Irena Kamińska [1] and Małgorzata Cieślak [1]

[1] Łukasiewicz Research Network - Textile Research Institute, Department of Chemical Textiles Technologies, 5/15 Brzezinska Street, 92-103 Lodz, Poland
[2] Łukasiewicz Research Network - Institute of Non-Ferrous Metals, 5 Sowińskiego Street, 44-100 Gliwice, Poland

Abstract: Silk as a protein fiber characterized by high biocompatibility, biodegradability, and low toxicity is mainly used as textile structures for various purposes, including biological applications. The key issue for the unlimited silk applicability as a modifier is to prepare its relevant form to cover or introduce to other materials. This study presents silk powder fabrication from *Bombyx mori* cocoons and non-dyed silk woven fabric through cryogenic milling. The cocoons were milled before and after the degumming process to obtain powders from raw structures and pure fibroin. The powder morphology and composition were analyzed using scanning electron microscopy and energy dispersive spectroscopy. The influence of the milling on the silk structure was studied using infrared and Raman spectroscopies indicating that silk powders retained dominant β-sheet structure. The powders were also analyzed by differential scanning calorimetry and thermogravimetric techniques. The thermal endothermic peak and onset temperature characteristic for silk decomposition shifted to the lower values for all powders indicating less thermal stability. However, the process was found to be an efficient way to obtain silk powders. The new milled form of silk can allow its introduction into different matrices or form coatings without using any harsh solvents, enriching them with new features and make more biologically friendly.

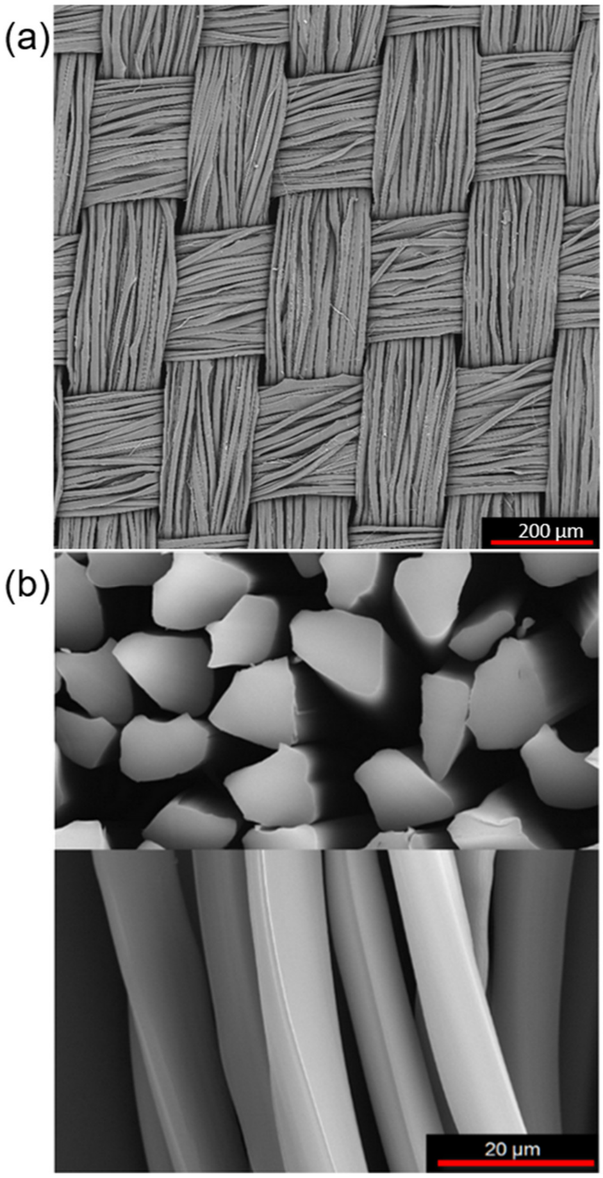

Figure S1. SEM images of (a) the top view, (b) cross-sectional view, and along the fibers of silk woven fabric.

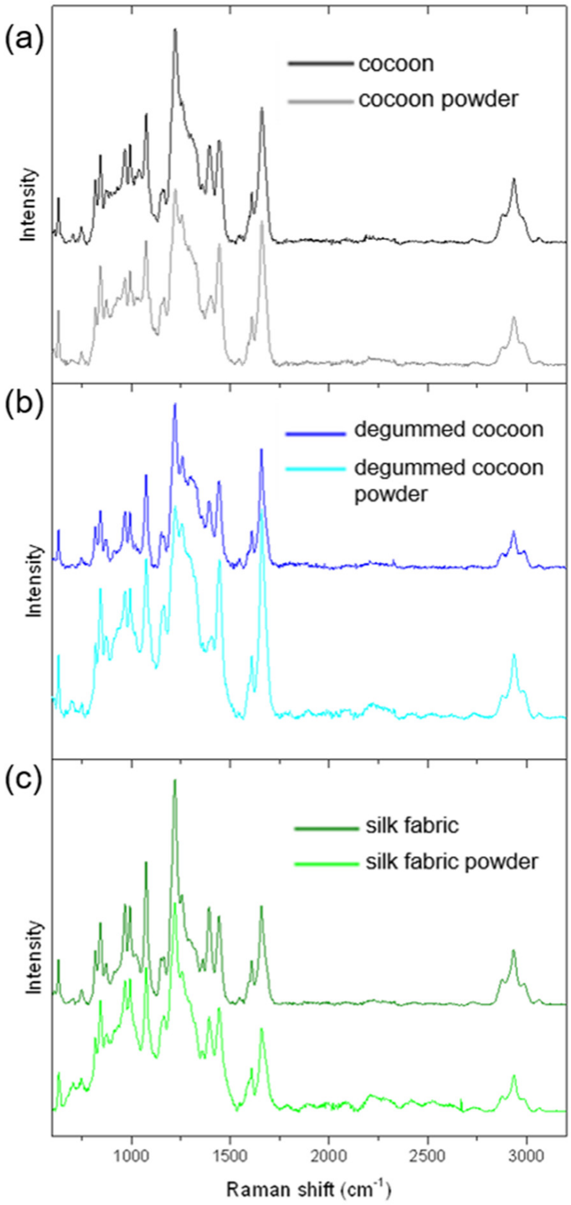

Figure S2. Raman spectra of (a) raw silk cocoons, (b) degummed cocoons, and (c) silk fabric, and silk powders from (a) raw cocoons, (b) degummed cocoons, and (c) silk fabric.

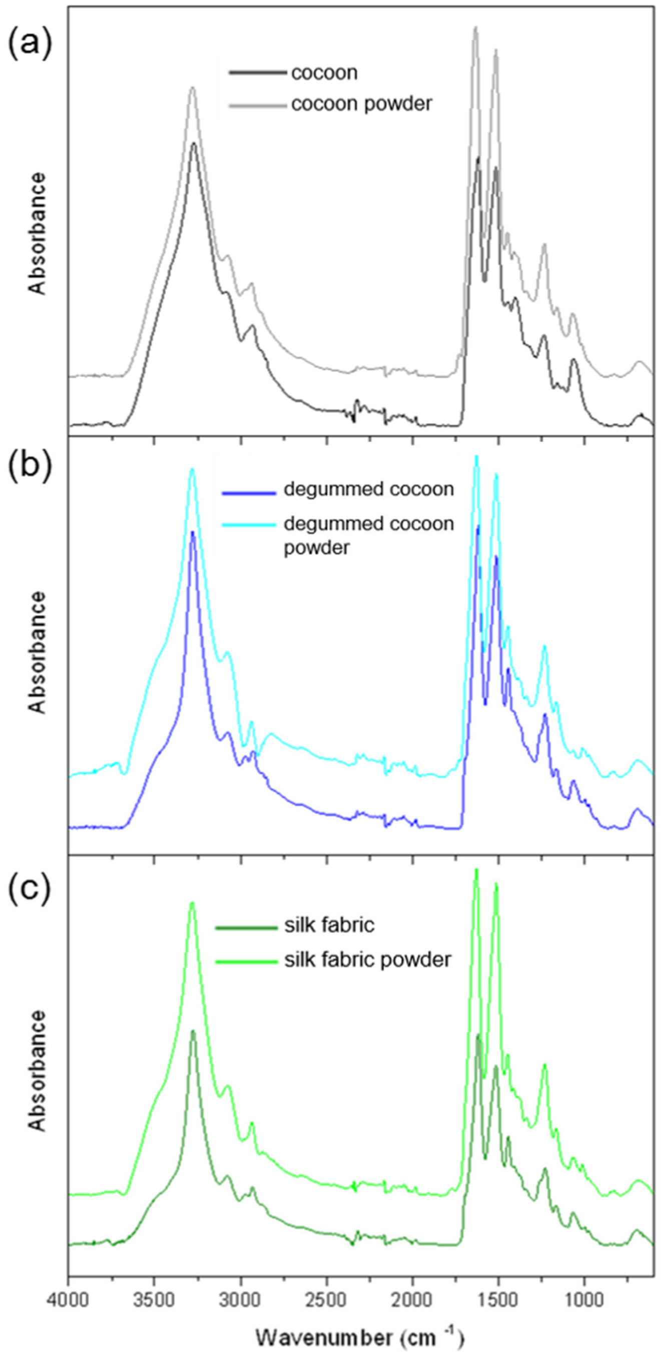

Figure S3. Infrared absorption spectra of (a) raw, and (b) degummed silk cocoons, (c) silk fabric, and silk powders from (a) raw cocoons, (b) degummed cocoons, and (c) silk fabric.

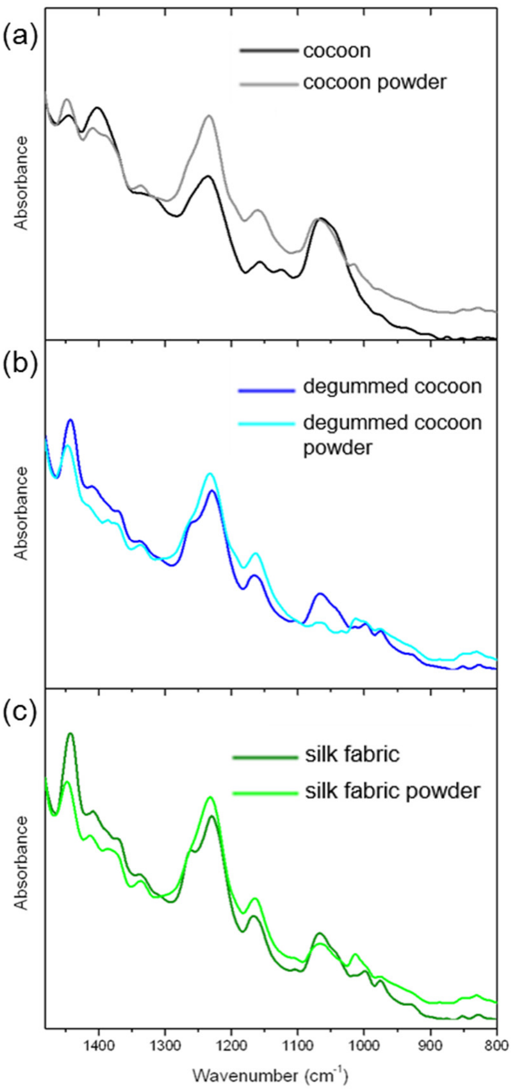

Figure S4. Infrared absorption spectra of (a) raw, and (b) degummed silk cocoons, (c) silk fabric, and silk powders from (a) raw cocoons, (b) degummed cocoons, and (c) silk fabric.

Table S1. The onset, and end temperatures, peak (T_d) of degradation process, and degradation enthalpy value (ΔH_{deg}) obtained from DSC analysis for raw, degummed silk cocoons and silk woven textile before and after cryogenic milling.

	**Onset temp. (ºC)**	**End temp. (ºC)**	**Peak T_d (ºC)**	**ΔH_{deg} (J/g)**
raw cocoons	270.9±1.1	337.3±0.9	317.2±0,8	228.5±2.8
raw cocoons - **powder**	268.9±1.5	335.3±1.5	312.7±0.6	257.7±3.7
degummed cocoons	272.8±1.4	342.9±1.4	320.3±0.6	278.9±3.3
degummed cocoons - **powder**	263.4±1.4	334.8±1.9	309.6±0.5	286.1±4.2
silk woven fabric	274.0±2.5	336.1±2.6	314.9±1.1	331.9±5.2
silk woven fabric - **powder**	258.6±2.6	334.0±2.1	301.8±0.8	333.7±4.9

Table S2. The onset, and end temperatures, and peak of degradation process, obtained from TG/DTG analysis for raw, degummed silk cocoons and silk woven textile before and after cryogenic milling.

	**Onset temp. (ºC)**	**End temp. (ºC)**	**Peak (ºC)**

raw cocoons	281.5±1.6	347.2±2.3	316.3±0.7
raw cocoons - **powder**	281.6±3.1	343.4±2.7	308.5±0.5
degummed cocoons	281.1±2.1	346.3±1.0	314.2±0.8
degummed cocoons - **powder**	281.6±0.9	343.4±1.1	308.2±0.7
silk woven fabric	279.6±0.9	342.1±1.1	309.6±0.8
silk woven fabric - **powder**	281.5±2.7	336.5±1.5	300.2±0.2
